# i.c.v. delivery of AAV9-synapsin-promoted caveolin-1 attenuates neuromuscular deficits and neuromuscular degeneration in hSOD1^G93A^ mice

**DOI:** 10.1016/j.omta.2026.201820

**Published:** 2026-07-27

**Authors:** Noriyuki Watanabe, Yumi Kawahori, Vinh Ta, Dongsheng Wang, Kenneth A. Ohgi, Hongxia Wang, Natalia Kleschevnikova, Eiichi Ishikawa, Brian P. Head

**Affiliations:** 1 Department of Anesthesiology, University of California, San Diego, 9500 Gilman Drive, La Jolla, San Diego, CA 92093, USA; 2 VA San Diego Healthcare System, 3350 La Jolla Village Drive, San Diego, CA 92161, USA; 3 Department of Neurosurgery, Institute of Medicine, University of Tsukuba, 1-1-1- Tennodai, Tsukuba, Ibaraki 305-8575, Japan

**Keywords:** AAV9-*SynCav1*, intracerebroventricular administration, amyotrophic lateral sclerosis, caveolin-1, motor neuron, neuroprotection

## Abstract

Practical and broad biodistributable gene delivery interventions are essential for advancing therapeutic strategies targeting neurodegenerative diseases such as amyotrophic lateral sclerosis (ALS). We previously demonstrated that subpial delivery of AAV9-synapsin-promoted caveolin-1 (*SynCav1*) afforded significant neuroprotective effects in mutant superoxide dismutase (SOD)-1-induced ALS pathology. However, subpial delivery is regionally restricted, technically challenging, and highly invasive. This study evaluated whether the intracerebroventricular (i.c.v.) route of administration (ROA), an alternative CNS delivery strategy less invasive than direct spinal cord injections, could achieve broader CNS biodistribution and produce functional or histological benefits in hSOD1^G93A^ mice. i.c.v. administration of AAV9-*SynCav1* achieved widespread Cav-1 overexpression in the motor cortex and spinal cord. *SynCav1*-treated male mice exhibited improved running wheel (RW) performance and better motor-evoked potentials. Immunofluorescence revealed attenuated degeneration of cholinergic motor neurons (MNs) in the cervical and lumbar ventral horn, as well as preserved diaphragm neuromuscular junction (NMJ) innervation in *SynCav1-*treated mice. These findings serve as preclinical proof of concept that i.c.v. delivery of AAV9-*SynCav1* can achieve CNS target engagement and produce selective functional and anatomical benefits in hSOD1^G93A^ mice.

## Graphical abstract

**Figure:**
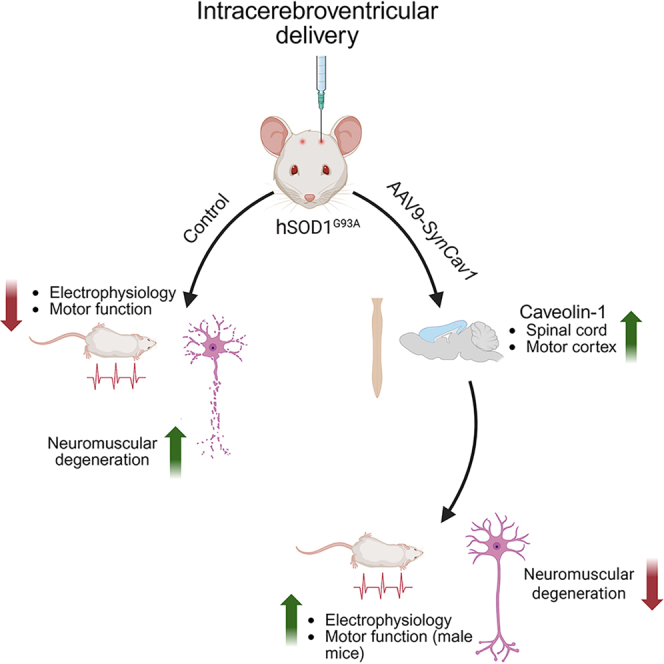

## Teaser

Intracerebroventricular (i.c.v.) delivery of AAV9-*SynCav1* provides broad CNS expression of caveolin-1, a membrane scaffolding protein, via a more accessible route than previously used subpial delivery. In an amyotrophic lateral sclerosis mouse model, this approach maintains motor unit integrity by attenuating electrophysiological deficits, motor neuron degeneration, and diaphragm denervation.

## Introduction

Amyotrophic lateral sclerosis (ALS) is a progressive neurodegenerative disease characterized by degeneration of upper (UMN) and lower motor neurons (LMNs) in the central nervous system (CNS). The incidence of ALS is 1.59 per 100,000 person-years, and the prevalence is 4.2–4.42 per 100,000 persons across the world.^1^^,^^2^ ALS incidence is higher in men than in women, with a ratio of about 1.6:1.^3^^,^^4^ Patients experience progressive paralysis throughout the body, with respiratory failure leading to early death in patients with ALS. Non-invasive ventilation is often the first choice to sustain quality of life (QOL) in the setting of respiratory function deterioration. However, as the symptoms progress, many patients face a difficult decision whether to pursue tracheostomy placement, which might further exacerbate the burden for caregivers. ALS exists in either familial (fALS) or sporadic (sALS) forms, with the latter accounting for more than 90% of ALS cases.^5^^,^^6^ A complex interplay of multiple factors contributes to ALS pathogenesis. Research into fALS has identified several causative gene mutations, including Cu/Zn superoxide dismutase (*SOD1*), *TARDBP* (also known as *TDP-43*), *FUS*, and *C9ORF72*.^5^^,^^7^ Mutations in *SOD1* were first reported as causative mutations for ALS in 1993, accounting for about 20% of fALS and 5% of sALS.^5^^,^^8^ Genetic interventions for fALS often focus on knocking down key genes like mutant *SOD1*.^9^^,^^10^^,^^11^ However, the etiology of the more prevalent sALS is highly complex, and successful ALS treatments likely require combinations of neuroprotective interventions. Therefore, interventions that provide neuroprotection and maintain the survival and neuromuscular function of MNs are in high demand.

One such neuroprotective protein is caveolin-1 (Cav-1), a cholesterol-binding and scaffolding protein within plasmalemmal microdomains termed membrane lipid rafts (MLRs). Within MLRs, Cav-1 anchors AMPAR, NMDAR, and TrkB, along with synaptic proteins necessary for synaptic transmission and neuroplasticity.^12^^,^^13^^,^^14^^,^^15^^,^^16^ Localization of these synaptic transmission components (neurotransmitters and receptors) with neuronal MLRs leads to increased production of cAMP, a second messenger that promotes neuronal growth and dendritic arborization.^12^^,^^17^ The transgenic (Tg) mouse model expressing the human mutant SOD-1 (hSOD1^G93A^) is a well-established preclinical model that recapitulates the progressive MN degeneration, muscle atrophy, paralysis, and early mortality observed in patients with ALS.^18^^,^^19^ Previous studies showed that direct *AAV9-*synapsin-promoted Cav-1 (*SynCav1*) delivery into the lumbar spinal cord of hSOD1^G93A^ mice increased survival of ventral horn MNs, improved motor performance and neuromuscular function, and extended lifespan in both male and female mice.^20^ However, direct spinal cord *AAV9-SynCav1* delivery only resulted in limited spinal cord biodistribution, thus confining neuron-targeted Cav-1 overexpression solely within the lumbar spinal cord. Moreover, subpial spinal cord injection carries a high risk of complications. To effectively target and mitigate widespread MN degeneration seen in ALS, broader spinal cord Cav-1 overexpression is desired.

The intracerebroventricular (i.c.v.) route of administration (ROA), on the other hand, is a less invasive, standard clinical procedure that circumvents the high surgical risks of subpial injections. Furthermore, i.c.v. delivery can achieve widespread CNS transduction, offering the possibility to target both upper and lower MNs in ALS.^21^^,^^22^ Therefore, the objective of this study was to determine whether broad spinal cord neuron-targeted Cav-1 overexpression could be achieved using a less invasive ROA and if this broader Cav-1 CNS biodistribution could produce neuroprotective effects comparable to those previously observed with direct delivery.

## Results

### i.c.v. administration of AAV9-*SynCav1* increases Cav-1 protein expression in the motor cortex, cervical, and lumbar spinal cords of male and female hSOD1^G93A^ mice

**Figure 1:**
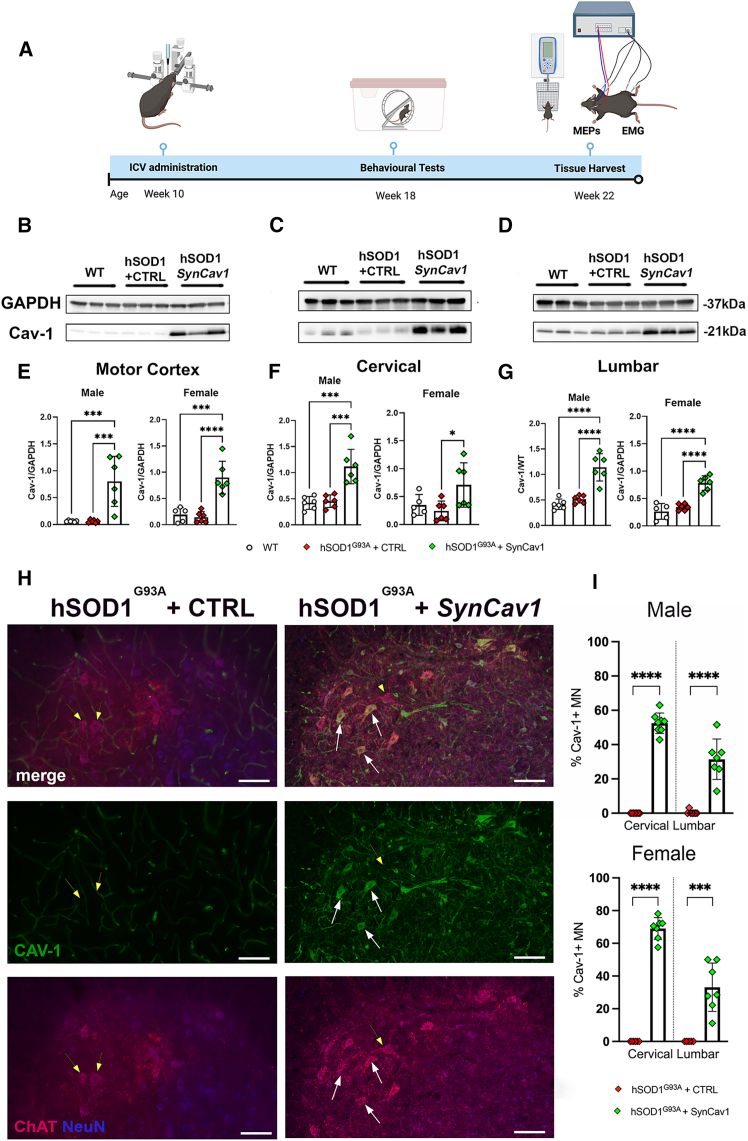
i.c.v. AAV9-*SynCav1* delivery increases Cav-1 protein expression in the motor cortex and the spinal cord in hSOD1^G93A^ mice (A) Schematic of experimental design and timeline (BioRender, https://BioRender.com/t168yx7). (B–D) Representative immunoblots on motor cortex (B), cervical (C), and lumbar cord (D), respectively. (E–G) Quantification of (B)–(D) for both sexes. Cav-1 was normalized to GAPDH. Each point represents one animal. Animal numbers for analysis: WT (male *n* = 6; female *n* = 5), hSOD1^G93A^ + CTRL (male *n* = 6 [6 saline]; female *n* = 6 [5 saline, 1 AAV9-SynRFP]), and hSOD1^G93A^ + *SynCav1* (male *n* = 6; female *n* = 6). (H) Representative images of ventral horn MN transduction in hSOD1^G93A^ control and hSOD1^G93A^*SynCav1* visualized with ChAT (red), NeuN (blue), and Cav-1 (green) co-staining. Yellow arrows denote examples of Cav-1-negative MNs; white arrows denote examples of Cav-1-positive MNs. Scale bar: 100 μm. (I) Quantification of Cav-1+ MNs in cervical and lumbar ventral horn for both sexes. Data are mean (SD). Statistical analysis was performed using one-way ANOVA with Tukey’s multiple comparisons test (E–G) or unpaired *t* test (I). Significance was assumed when *p* < 0.05. ∗*p* < 0.05, ∗∗*p* < 0.01, ∗∗∗*p* < 0.005, and ∗∗∗∗*p* < 0.0001.

To evaluate i.c.v. AAV9-*SynCav1* delivery in a preclinical ALS model, hSOD1^G93A^ mice received AAV9-*SynCav1* or control treatment via i.c.v. injection at 10 weeks of age. Voluntary running wheel (RW) was performed at 18 weeks of age, grip strength (GS) test, and electrophysiology at 22 weeks of age, and mice were sacrificed shortly after for biochemical and histological assays (Figure 1A). To validate transgene expression, immunoblot was used to compare protein levels in bulk tissue between hSOD1^G93A^ + control (CTRL) mice and hSOD1^G93A^ + *SynCav1* mice. Tg-negative littermates were included as a reference for basal expression levels. *SynCav1* treatment produced robust Cav-1 overexpression across ALS-relevant CNS regions, including the motor cortex, cervical spinal cord, and lumbar spinal cord. In the motor cortex, *SynCav1* significantly elevated Cav-1 protein expression in both males and females compared to the CTRL group (*p* < 0.001 for each comparison; Figures 1B and 1E). In the cervical spinal cord, Cav-1 protein expression was significantly higher in the *SynCav1*-treated mice than in the CTRL mice in both males and females (*p* < 0.001 and *p* = 0.012, respectively; Figures 1C and 1F). Similarly, in the lumbar spinal cord, *SynCav1* treatment significantly increased Cav-1 protein expression compared to their respective CTRL groups in both sexes (*p* < 0.001 for each; Figures 1D and 1G). Because spinal MNs (SMNs) are a major site of degeneration in ALS, we next assessed whether AAV9*-SynCav1* increased Cav-1 expression specifically in ChAT+/NeuN+ MNs using immunofluorescence (Figure 1H). Cav-1 expression was minimal in hSOD1^G93A^ + CTRL mice, whereas *SynCav1* produced prominent Cav-1 overexpression within ChAT+/NeuN+ MNs. Quantification showed substantial MN Cav-1 expression in the cervical spinal cord of both male and female hSOD1^G93A^ + *SynCav1* mice (*p* < 0.001 in both sexes), with lower but detectable transduction in the lumbar spinal cord (*p* = 0.0097 in males and *p* < 0.001 in females; Figure 1I). Together, these data suggest that i.c.v. delivery of AAV9-*SynCav1* achieved robust Cav-1 protein expression, not only in the motor cortex but also in vulnerable ChAT+/NeuN+ SMN populations.

### *SynCav1*-treated hSOD1^G93A^ male mice exhibit better voluntary RW performance and muscle electrophysiological properties

**Figure 2:**
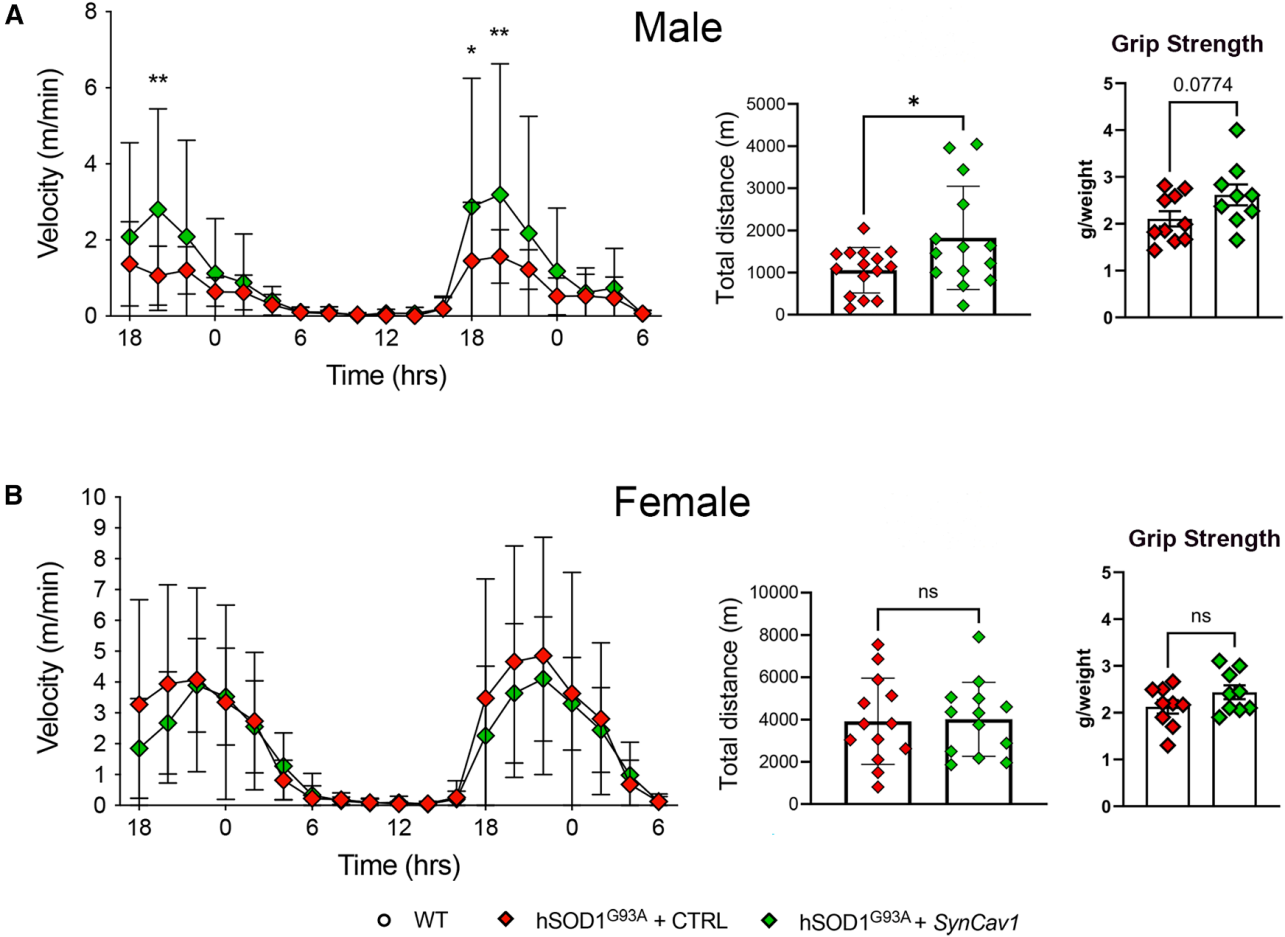
*SynCav1*-treated hSOD1^G93A^ male mice show maintained RW motor performance at 18 weeks of age and a trend toward GS at 22 weeks of age (A and B) Left to right: average voluntary RW velocity (m/min) per 2 h over 36 h, total voluntary RW distance over 36 h, and GS test in male (A) and female (B) hSOD1^G93A^ mice treated with control or *SynCav1*. Data are presented as mean (SD). For RW: hSOD1^G93A^ + CTRL (male *n* = 15 [11 saline, 4 AAV9-SynRFP]; female *n* = 13 [10 saline, 3 AAV9-SynRFP]) and hSOD1^G93A^ + *SynCav1* (male *n* = 14; female *n* = 13). For GS: hSOD1^G93A^ + CTRL (male *n* = 9 [7 saline, 2 AAV9-SynRFP]; female *n* = 11 [8 saline, 3 AAV9-SynRFP]) and hSOD1^G93A^ + *SynCav1* (male *n* = 9; female *n* = 10). Statistical analysis was performed using two-way ANOVA followed by Bonferroni’s multiple comparisons test for RW velocity and unpaired *t* test for total wheel distance and GS. NS indicates no significance (*p* > 0.05), and significance was assumed when *p* < 0.05. ∗∗*p* < 0.01.

To determine *SynCav1*’s effects on neuromuscular function, we performed voluntary RW, GS, motor-evoked potentials (MEPs), and electromyography (EMG) in *SynCav1*-treated hSOD1^G93A^ mice. Because our previous reports showed that hSOD1^G93A^ mice present with prominent deficits in RW performance compared to wild-type (WT) mice,^15^^,^^20^ we compared only the hSOD1^G93A^ groups to remove animal-use redundancy. In males only, hSOD1^G93A^ + *SynCav1* mice demonstrated longer total wheel distance (*p* = 0.045) and greater velocity on RW compared to hSOD1^G93A^ + CTRL mice (*p* = 0.0055–0.045; Figure 2A). Using the GS test, male hSOD1^G93A^ + *SynCav1* mice exhibited a non-significant trend toward higher GS compared to hSOD1^G93A^ + CTRL mice (*p* = 0.074; Figure 2A). For females, no difference in RW or GS between the CTRL and treatment groups was detected (Figure 2B).

**Figure 3:**
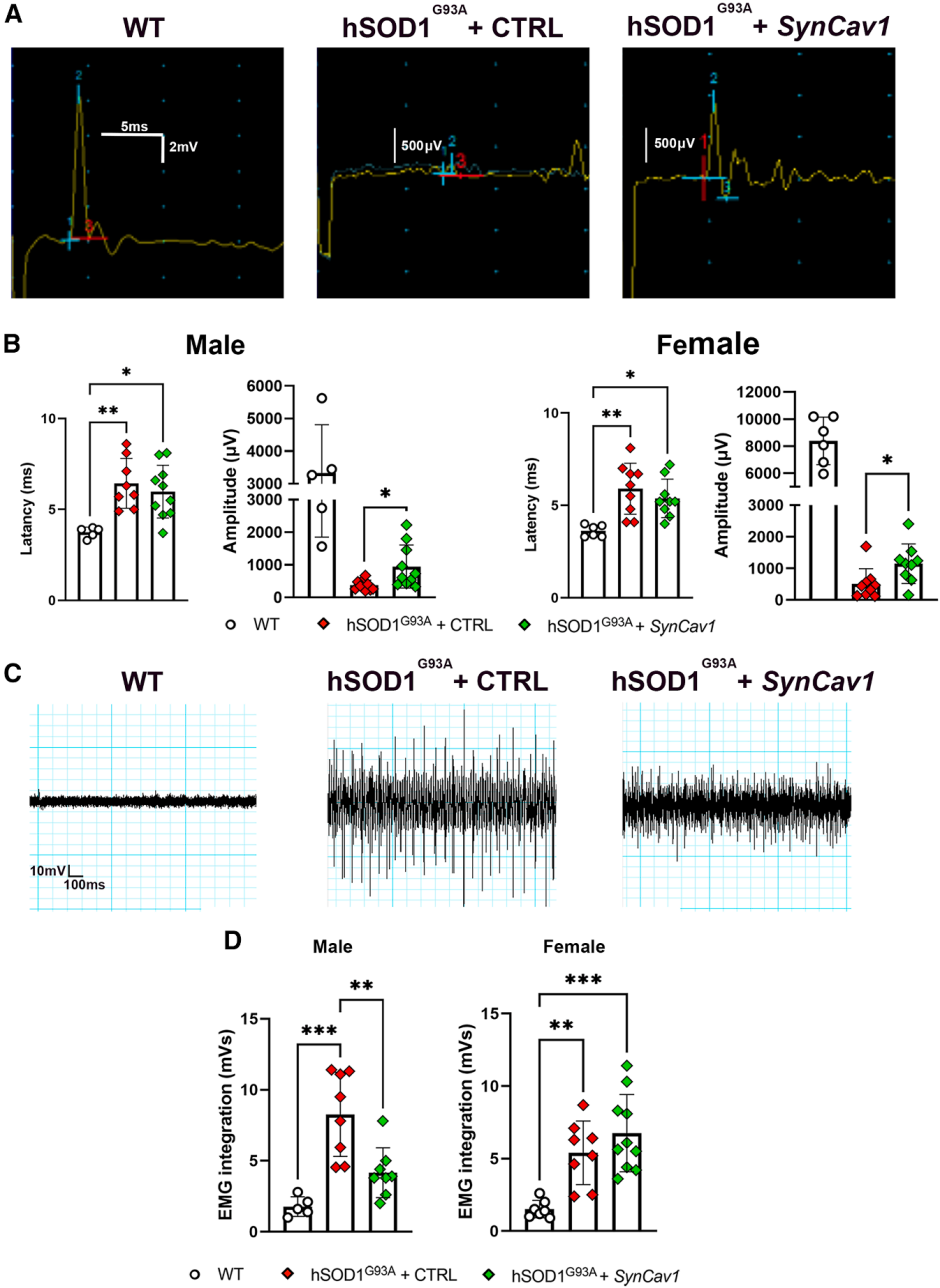
*SynCav1* attenuates muscle electrophysiological abnormalities in late-stage symptomatic hSOD1^G93A^ mice at 22 weeks of age (A) Annotated representative pictures of MEPs for WT, SOD1-CTRL, and SOD1-*SynCav1.* MEP onset is denoted by 1, peak amplitude is denoted by 2, and end of response is denoted by 3. (B) Quantification of MEP latency and amplitude in the triceps muscles. (C) Representative resting EMG traces from the gastrocnemius muscle. (D) Integration of resting EMG amplitudes. Data are presented as mean (SD). For MEPs: WT (male *n* = 5; female *n* = 6), hSOD1^G93A^ + CTRL (male *n* = 8 [8 saline]; female *n* = 9 [6 saline, 3 AAV9-SynRFP]), and hSOD1^G93A^ + *SynCav1* (male *n* = 10; female *n* = 9). For EMG: WT (male *n* = 5; female *n* = 7), hSOD1^G93A^ + CTRL (male *n* = 8 [8 saline]; female *n* = 8 [7 saline, 1 AAV9-SynRFP]), and hSOD1^G93A^ + *SynCav1* (male *n* = 8; female *n* = 9). Statistical analysis was performed using one-way ANOVA followed by post hoc Tukey’s comparisons test for MEP latency and EMG amplitude, followed by Welch’s *t* test for MEP amplitude (male) and unpaired *t* test (female). Significance was assumed when ∗*p* < 0.05, ∗∗*p* < 0.01, and ∗∗∗*p* < 0.005.

To determine the functional benefit of *SynCav1* treatment, we assessed the integrity of the motor system by measuring MEPs (latency and amplitude) and EMG integration (Figure 3A). MEPs revealed that both hSOD1^G93A^ groups, CTRL and *SynCav1*, had significantly longer latency and prominently lower amplitude than WT mice, while *SynCav1*-treated hSOD1^G93A^ mice exhibited increased amplitude in both sexes. The increase in MEP amplitude was statistically significant in male (*p* = 0.025) and female (*p* = 0.028) hSOD1^G93A^ mice that received *SynCav1* compared to the hSOD1^G93A^ + CTRL group (Figure 3B). There was no difference in MEP latency between hSOD1^G93A^ + CTRL and hSOD1^G93A^ + *SynCav1* mice (Figure 3B).

We next quantified the integrated EMG signal. In males, hSOD1^G93A^ + CTRL mice showed significantly higher integrated EMG amplitudes compared to both WT mice (*p* < 0.001) and hSOD1^G93A^ + *SynCav1* mice (*p* = 0.0013). Importantly, the amplitudes in hSOD1^G93A^ + *SynCav1* mice did not differ significantly from those in WT mice (Figure 3D). In females, EMG amplitude was significantly elevated in both hSOD1^G93A^ groups compared to WT (CTRL vs. WT: *p* = 0.005; SynCav1 vs. WT: *p* < 0.001), but amplitudes did not differ between hSOD1^G93A^ + CTRL and hSOD1^G93A^ + *SynCav1* groups (Figure 3D). Collectively, these data suggest that *SynCav1* treatment improves select motor system functions, increasing MEP amplitude for both sexes and providing more apparent functional and EMG benefits in males.

### AAV9*-SynCav1* increases Cav-1 expression in MNs and partially attenuates MN pathology

**Figure 4:**
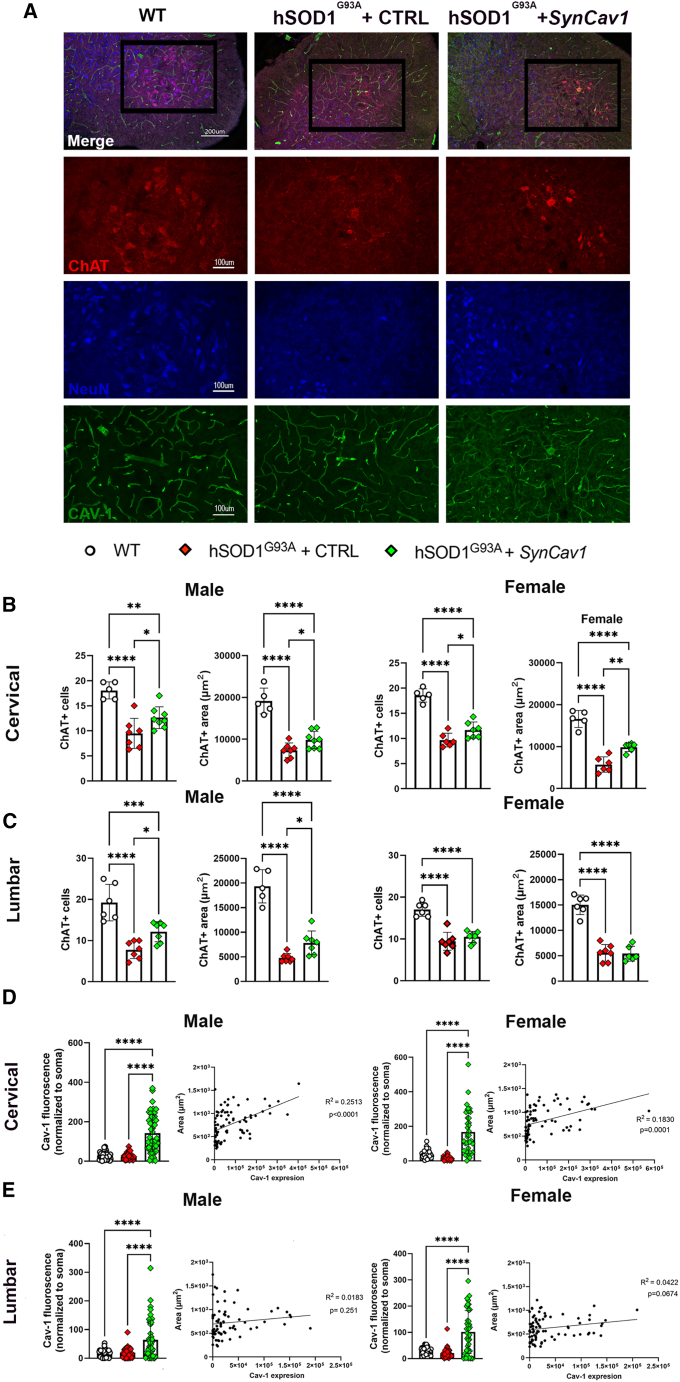
*SynCav1* partially mitigates MN degeneration in cervical and lumbar ventral horns in late-stage symptomatic SOD1^G93A^ mice at 22 weeks of age (A) Representative images of MNs in the ventral horn of the cervical spinal cord identified by ChAT (red), NeuN (blue), and Cav-1 (green) immunofluorescence. Scale bar: 100 μm. (B and C) Quantification of MN number and total ChAT+ area (μm^2^) in the cervical (B) and lumbar (C) regions for both sexes. (D and E) Quantification of Cav-1 intensity in MNs along with correlation analysis of Cav-1 fluorescence and MN area in cervical (D) and lumbar (E) regions for both sexes. Each point represents an MN for (D) and (E). Data are presented as mean (SD). Animal numbers for cervical histological analysis: WT (male *n* = 5; female *n* = 5), hSOD1^G93A^ + CTRL (male *n* = 7 [7 saline]; female *n* = 6 [6 saline]), and hSOD1^G93A^ + *SynCav1* (male *n* = 8; female *n* = 7). For lumbar histological analysis: WT (male *n* = 6; female *n* = 6), hSOD1^G93A^ + CTRL (male *n* = 7 [6 saline, 1 AAV9-SynRFP]; female *n* = 7 [5 saline, 2 AAV9-SynRFP]), and hSOD1^G93A^ + *SynCav1* (male *n* = 7; female *n* = 7). Statistical analysis was performed using one-way ANOVA with Fisher’s LSD multiple comparisons test, followed by Pearson’s r correlation analysis for linear regression. Significance was assumed when *p* < 0.05, ∗∗*p* < 0.01, ∗∗∗*p* < 0.005, and ∗∗∗∗*p* < 0.0001.

Having observed selective improvements in motor performance and neuromuscular electrophysiological outcomes, we next assessed whether these functional effects were accompanied by preservation of SMNs. To evaluate this, we visualized and quantified ChAT+/NeuN+ MNs and ChAT+ MN area in the cervical and lumbar spinal cord of hSOD1^G93A^ CTRL and *SynCav1* mice (Figure 4A). In the cervical ventral horn, both hSOD1^G93A^ groups (CTRL and *SynCav1*) exhibited a significant reduction in the number of ChAT+ MNs and in MN soma area per ventral horn compared to WT mice (Figure 4B). However, *SynCav1* treatment mitigated MN loss: the number of ChAT+ MNs was significantly higher in the *SynCav1*-treated group compared to the hSOD1^G93A^ + CTRL group in males (*p* = 0.022) and females (*p* = 0.027) (Figure 4B). This effect extended to MN morphology in that the total ChAT+ MN area per ventral horn, a measure of overall MN soma size, was significantly larger in hSOD1^G93A^ + *SynCav1* mice than in hSOD1^G93A^ + CTRL in both sexes, suggesting attenuated MN atrophy (*p* = 0.047 in males and *p* < 0.001 in females; Figure 4B).

Similarly, in the lumbar ventral horn, both male and female hSOD1^G93A^ mice showed reduced ChAT+ MN number and total ChAT+ MN area compared with WT mice (Figure 4C). In males, hSOD1^G93A^ + *SynCav1* mice showed a significantly higher number of ChAT+ MNs compared with hSOD1^G93A^ + CTRL mice (*p* = 0.017), as well as higher total ChAT+ MN area (*p* = 0.027; Figure 4C). In females, no significant differences between hSOD1^G93A^ + CTRL and hSOD1^G93A^ + *SynCav1* mice were detected in the lumbar spinal cord.

For both the cervical and lumbar regions, Cav-1 fluorescence intensity normalized to MN area was significantly elevated in the hSOD1^G93A^ + *SynCav1* group compared to the hSOD1^G93A^ + CTRL groups for both sexes (*p* < 0.001 for both sexes; Figures 4D and 4E). Simple linear regression correlation analysis of the cervical region revealed a significant positive correlation between Cav-1 fluorescence levels and MN area of the cervical spinal cord, indicating that higher Cav-1 expression was associated with larger MN soma area (*p* < 0.001 for both sexes; Figure 4D). In the lumbar spinal cord, this relationship trended toward significance in females but was not significant in males (*p* = 0.067 in females and *p* = 0.25 in males; Figure 4E). Overall, these findings suggest that i.c.v.-delivered AAV9-*SynCav1* produces modest, region- and sex-dependent effects on SOD1-induced SMN degeneration.

### AAV9*-SynCav1* mitigates diaphragm muscle neuromuscular junction denervation in hSOD1^G93A^ mice in both sexes

**Figure 5:**
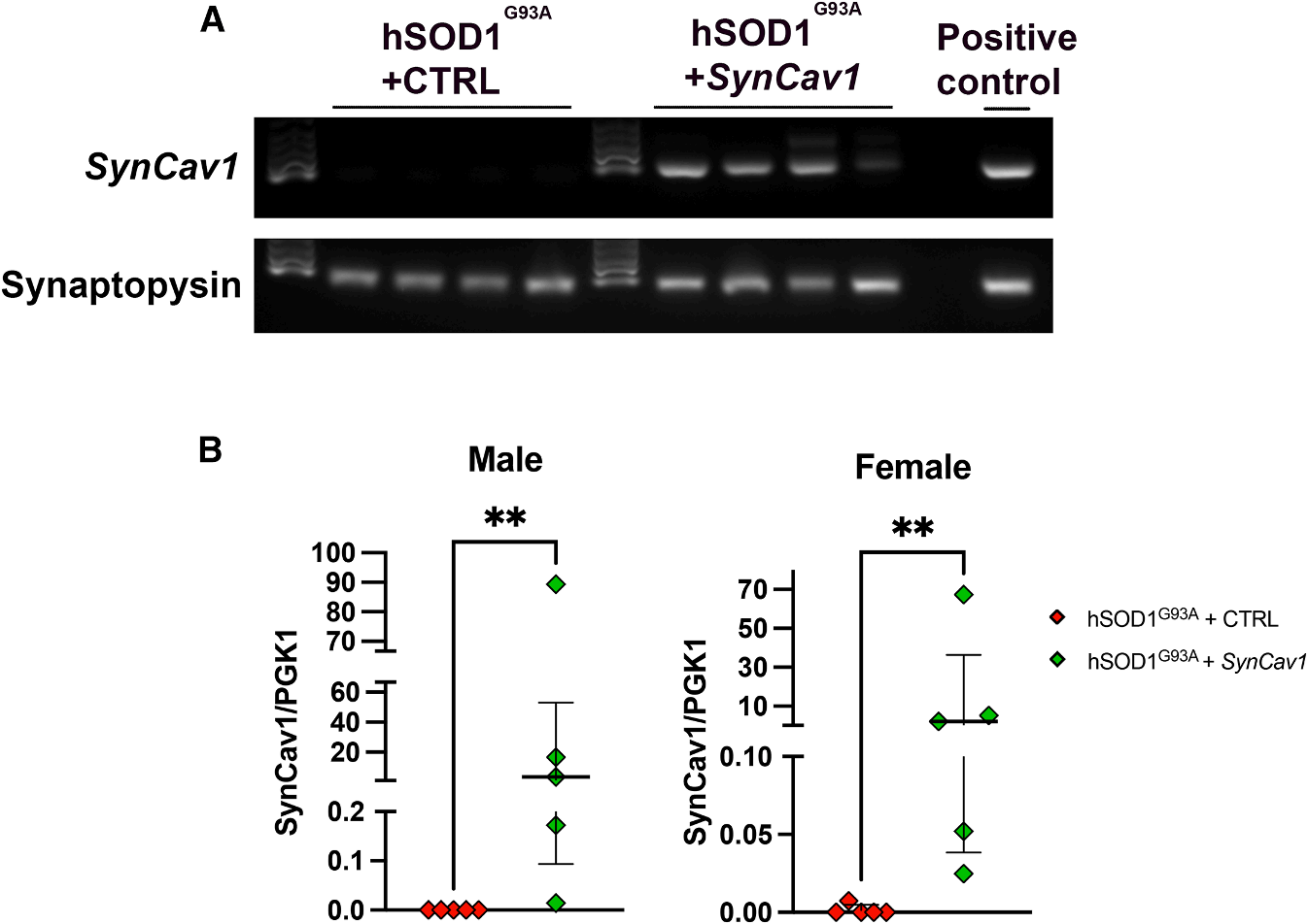
i.c.v. AAV9-*SynCav1* delivery produces peripheral neuromuscular target engagement in diaphragm muscles. hSOD1^G93A^ mice received i.c.v. AAV9-*SynCav1* (WPRE), and diaphragm tissues were harvested for RT-qPCR and agarose gel validation (A) Representative agarose gels showing *SynCav1* and synaptophysin transcripts in diaphragm tissue. Spinal cord tissue from validated *SynCav1*-treated mice was used as positive controls. (B) RT-qPCR quantification of *SynCav1* expression normalized to PGK1. Animal numbers for analysis: hSOD1^G93A^ + CTRL (male *n* = 5 [4 saline, 1 AAV9-SynRFP]; female *n* = 5 [4 saline, 1 AAV9-SynRFP]) and hSOD1^G93A^ + *SynCav1* (male *n* = 5; female *n* = 5). Data are presented as mean (SD). Statistical analyses were performed using Mann-Whitney tests. ∗∗*p* < 0.01.

To assess whether AAV9-*SynCav1* transgene expression could be detected in peripheral motor targets, we next focused on the diaphragm. Because disease progression in this mouse model advances from the lower spinal cord,^15^ the diaphragm—an effector muscle highly relevant to ALS respiratory pathology receiving phrenic motor innervation from the upper cervical spinal cord—presumably undergoes degeneration at the late stage. Agarose gel electrophoresis further confirmed detection of *SynCav1* transcript in diaphragm samples, with synaptophysin used as a neuromuscular junction (NMJ)-associated marker (Figure 5A). *SynCav1* mRNA transcript was detectable in diaphragm tissue from hSOD1^G93A^ + *SynCav1* mice, although expression levels varied between animals (Figure 5B).

**Figure 6:**
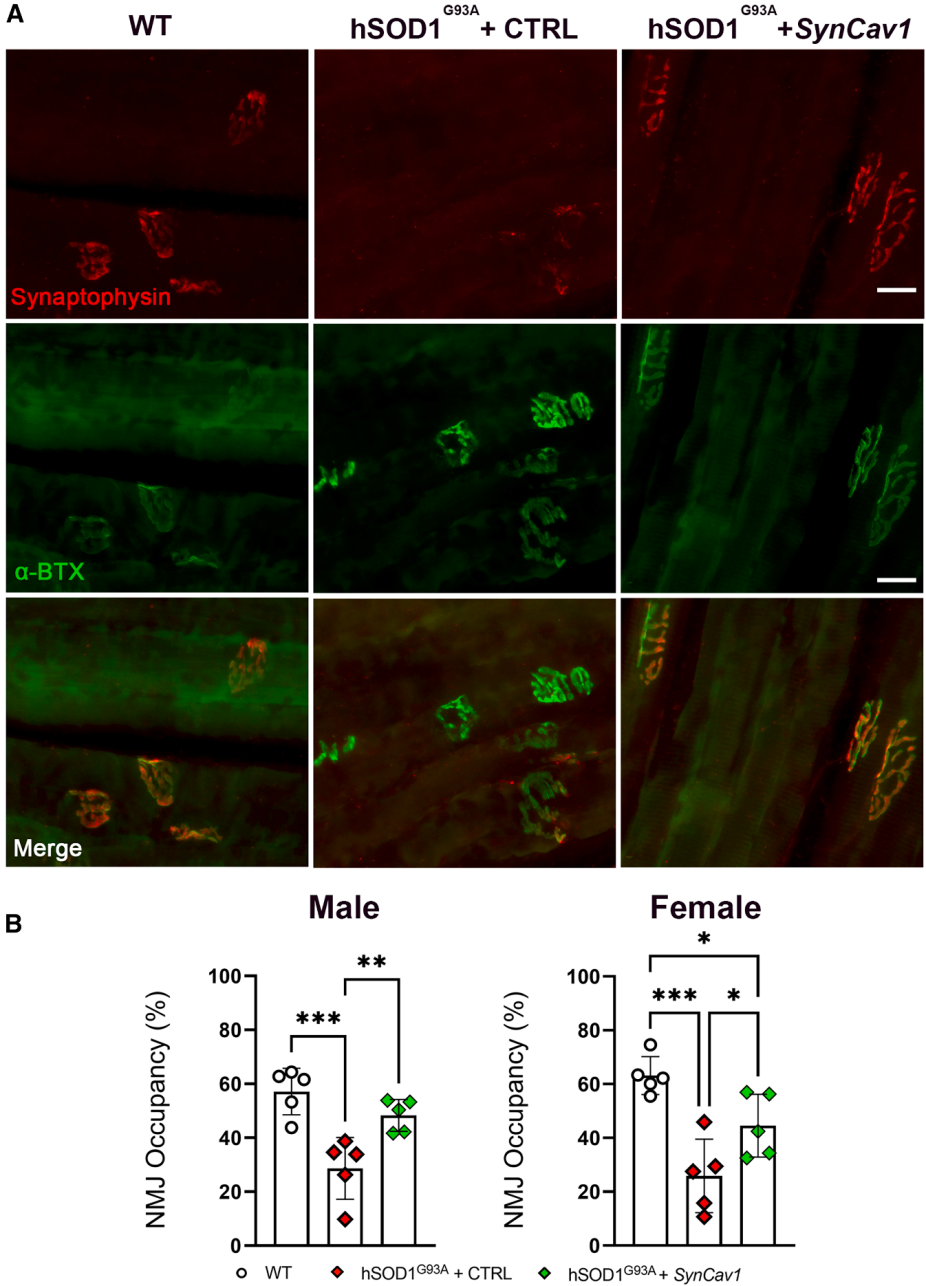
*SynCav1* preserves NMJ innervation in the diaphragm of hSOD1^G93A^ mice at 22 weeks of age (A) Representative images of diaphragm NMJ with α-bungarotoxin (green) and synaptophysin (red). Scale bar: 20 μm. (B) Quantification of NMJ occupancy, defined as the percentage of the postsynaptic α-bungarotoxin area overlapping with presynaptic synaptophysin, was analyzed. Animal numbers for analysis: WT (male *n* = 5; female *n* = 5), hSOD1^G93A^ + CTRL (male *n* = 5 [4 saline, 1 AAV9-SynRFP]; female *n* = 5 [3 saline, 2 AAV9-SynRFP]), and hSOD1^G93A^ + *SynCav1* (male *n* = 5; female *n* = 5). Data are presented as mean (SD). Statistical analysis was performed using one-way ANOVA with Fisher’s LSD multiple comparisons test. Significance was assumed when ∗*p* < 0.05 and ∗∗∗*p* < 0.005.

Given that *SynCav1* treatment partially preserved cervical MNs and *SynCav1* mRNA was detected in diaphragm tissue, we next evaluated whether these effects were accompanied by changes in diaphragm NMJ morphology at 22 weeks. hSOD1^G93A^ + CTRL mice, compared to WT mice, had significantly decreased NMJ synaptophysin occupancy in both sexes, indicating progressive axonal denervation, whereas hSOD1^G93A^ + *SynCav1* mice displayed significantly greater NMJ occupancy compared to hSOD1^G93A^ + CTRL mice in both sexes (*p* = 0.0047 in males and *p* = 0.021 in females; Figures 6A and 6B). No difference between WT and hSOD1^G93A^ + *SynCav1* was detected for males, while female hSOD1^G93A^ + *SynCav1* mice displayed significantly less innervation than the WT group (*p* = 0.021). These findings confirm *SynCav1* target engagement in upper limb and diaphragm regions and suggest downstream preservation of diaphragm NMJ integrity in male mice, with partial preservation in female mice.

### Analysis of sex as a biological variable

To assess potential sex-dimorphic responses to *SynCav1* treatment, we analyzed sex as a biological variable across all major behavioral, electrophysiological, and histological endpoints using a two-way analysis of variance (ANOVA; treatment group × sex) (Table 1). While several readouts—including Cav-1 expression throughout the CNS, RW total distance, and diaphragm NMJ innervation—demonstrated no significant sex × treatment interaction, the analysis revealed distinct sex-specific treatment benefits in the lower motor circuitry. Specifically, the *SynCav1*-associated therapeutic effect was significantly more pronounced in males for lower-limb resting EMG amplitude (*p* = 0.0149) and lumbar MN soma area (*p* = 0.0395). Conversely, *SynCav1*-mediated increases in Cav-1 fluorescence within lumbar MNs were significantly more pronounced in females (*p* < 0.001).

**Table 1:** Two-way ANOVA of sex as a biological variable across major endpoints

Figure	Endpoint	Sex effect *p* value	Group effect *p* value	Sex × group interaction *p* value	Sex × group interaction significant?	Interpretation
Figure 1E	motor cortex Cav-1 expression	0.2184	<0.0001	0.973	no	no significant sex × group interaction
Figure 1F	cervical spinal cord Cav-1 expression	0.0106	<0.0001	0.2725	no	no significant sex × group interaction
Figure 1G	lumbar spinal cord Cav-1 expression	<0.0001	<0.0001	0.1871	no	no significant sex × group interaction
Figure 2	RW total distance	<0.0001	0.2836	0.4004	no	no significant sex × group interaction
Figure 2	grip strength	0.6427	0.0226	0.5658	no	no significant sex × group interaction
Figure 3B	MEP latency	0.2425	<0.0001	0.8563	no	no significant sex × group interaction
Figure 3B	MEP amplitude	<0.0001	<0.0001	<0.0001	yes	significant sex × group interaction driven by WT sex difference; no evidence of sex-dependent SynCav1 effect (Tukey post hoc, *p* = 0.6296)
Figure 3D	EMG amplitude	0.7795	<0.0001	0.0028	yes	SynCav1-associated effect appeared more pronounced in males (Tukey post hoc, *p* = 0.0149)
Figure 4B	cervical MN count	0.8854	<0.0001	0.6273	no	no significant sex × group interaction
Figure 4B	cervical MN area	0.3696	<0.0001	0.1122	no	no significant sex × group interaction
Figure 4C	lumbar MN count	0.0466	<0.0001	0.2947	no	no significant sex × group interaction
Figure 4C	lumbar MN area	0.0054	<0.0001	0.012	yes	SynCav1-associated effect appeared more pronounced in males (Tukey post hoc, *p* = 0.0395)
Figure 4D	cervical MN Cav-1 fluorescence	0.2881	<0.0001	0.318	no	no significant sex × group interaction
Figure 4E	lumbar MN Cav-1 fluorescence	0.0036	<0.0001	0.0399	yes	SynCav1-associated effect appeared more pronounced in females (Tukey post hoc, *p* = 0.0003)
Figure 6B	diaphragm NMJ innervation	0.9626	<0.0001	0.5006	no	no significant sex × group interaction

ANOVA, analysis of variance; Cav-1, caveolin-1; EMG, electromyography; MEP, motor-evoked potential; MN, motor neuron; NMJ, neuromuscular junction; WT, wild type.

## Discussion

The present study examined the potential neuroprotective effects of AAV9-*SynCav1* gene delivery in hSOD1^G93A^ mice following i.c.v. ROA. i.c.v. delivery of *SynCav1* achieved broad Cav-1 transduction throughout the motor cortex and spinal cord, attenuated lower MN degeneration, and reduced disease-associated resting EMG amplitude while maintaining MEP amplitude. This is an important finding because i.c.v. administration achieved the beneficial effect on the cervical spinal cord without direct spinal cord injection. Subpial gene delivery would carry a tremendous risk because of critical, life-maintaining functions of the cervical spinal cord. To transduce target genes using AAV, there are several ROAs available, including subpial, systemic, and intra-cerebrospinal fluid (CSF) administration. For subpial administration, although it enables robust transgene expression in SMNs in rodent models,^11^^,^^20^ its translation to the clinic remains immensely challenging due to the inherent procedural risk of iatrogenic neurological damage. Since the transduction area is limited, it may require multiple injection sites across the spinal cord, which will also increase the postoperative complication risks. While systemic administration could be achieved through either intravenous or intra-arterial injection, there are several issues, including off-target effects in peripheral organs, especially the liver, and mitigating effects from pre-existing circulating anti-AAV-neutralizing antibodies.^23^^,^^24^^,^^25^ Despite promising technological advancements in the AAV field regarding the use of suitable AAVs that can target human receptors on the blood-brain barrier (BBB) for systemic delivery to the CNS, they are still in the preclinical stage and have yet to be FDA approved.^26^ Intra-CSF administration, which includes intrathecal (i.t.) and i.c.v., is relatively safe and enables broader gene transduction in the CNS^27^^,^^28^ with less off-target tissue effects and anti-AAV-neutralizing antibodies.^29^^,^^30^ Even though i.t. is highly translatable and effective for local spinal targets, i.c.v. delivery provides more direct access to the cerebral ventricles, enabling the broad CNS transduction necessary to target both upper and lower MNs in ALS.

Our previous report showed that direct spinal cord ROA of *AAV9-SynCav1* resulted in over a 6-fold increase in Cav-1 expression in MNs in the lumbar spinal cord,^20^ whereas the current i.c.v. approach produced a 2- to 3-fold change in the cervical cord. The lower MN Cav-1 overexpression may be sufficient to evoke neuroprotection, as demonstrated by the better RW performance and EMG findings in the male hSOD1^G93A^ mice at 22 weeks of age. Because resting EMG under anesthesia captures spontaneous denervation activity rather than voluntary muscle activation, the elevated integrated EMG observed in hSOD1^G93A^ CTRL mice is consistent with previous studies showing that spontaneous fibrillations and fasciculations emerge with motor unit denervation in ALS models.^3^^,^^4^^,^^5^ *SynCav1* treatment mitigated this pathological increase in male mice, supporting preservation of NMJ integrity. As MEPs are considered reliable indicators of upper MN involvement in human research using transcranial magnetic stimulation,^31^^,^^32^ our improvement in MEPs implies that this treatment might have a neuroprotective effect not only in LMNs but also in UMNs. This warrants future investigation of the corticospinal tract.

The present findings show that Cav-1 overexpression significantly improved MEP amplitudes in both sexes and improved lower-limb EMG and RW motor performance in male mice only. These sex differences related to motor and neuromuscular function could be explained by the biochemical finding that the neuroprotective effect of *SynCav1* on MNs in the lumbar ventral horn was observed only in males (Figure 4C). Further two-way ANOVA revealed an intriguing paradox regarding sex-specific therapeutic efficacy. The treatment consistently enhanced central motor conduction (MEP amplitude) and preserved diaphragm NMJ morphology independent of sex. However, within the lower motor circuitry, we observed an inverse relationship between lumbar transgene expression and functional therapeutic benefit. Specifically, *SynCav1*-mediated increases in Cav-1 fluorescence within lumbar MNs were significantly more pronounced in female mice. Paradoxically, despite this superior target engagement in the female lumbar cord, the actual therapeutic benefits— the attenuation of both pathological resting EMG amplitude and motor decline (RW performance), alongside the mitigation of lumbar MN soma atrophy—were more robust in males (Table 1).

This unexpected discrepancy suggests that the neuroprotective properties afforded by Cav-1 overexpression are not solely dictated by absolute transgene expression levels but also depend on sex-specific biological sensitivities. While higher AAV9 transduction efficiency in females has been previously noted following other ROAs,^33^ our functional and histological data indicate that male lower MNs may be inherently more responsive to Cav-1-mediated benefit. Alternatively, this might reflect baseline physiological variances in ALS disease progression or sex-based differences in immune responses to AAV-mediated gene therapy.^34^ Ultimately, these findings underscore the critical importance of analyzing sex as a biological variable, revealing that robust vector delivery does not universally equate to superior therapeutic efficacy across both sexes.

Respiratory failure is the inevitable terminal cause for patients with ALS; as the disease progresses, the loss of phrenic nerve function (which projects from the cervical [C3–C5] spinal cord) largely contributes to diaphragm muscle atrophy.^35^ Since respiratory function is vital to maintaining life, it raises several issues, including the use of mechanical ventilation.^36^ In the current study, with the RT-qPCR detection of *SynCav1* mRNA in the diaphragm of the treated hSOD1^G93A^ mice combined with the preservation of NMJ occupancy within the diaphragm in both sexes, the i.c.v. ROA has the potential to evoke neuroprotection, preserve NMJs within the diaphragm, and slow disease progression. Approaches using gene therapies that target the diaphragm often rely on highly invasive methods,^37^^,^^38^ which are unsuitable from a translational standpoint. Our demonstration that neuron-targeted Cav-1 overexpression can maintain phrenic nerve innervation via a relatively less invasive i.c.v. ROA, suggesting a potential therapeutic strategy to mitigate decline of respiratory function in ALS. Future studies will need to test if i.c.v. *SynCav1* delivery can preserve respiratory function in ALS models.

There are several limitations to the current study. Firstly, i.c.v. administration in mice might result in varied distribution due to differences in CSF circulation from disease-related anatomical changes. Previous work in non-human primates, i.c.v. administration resulted in a strong distribution around the brain with transduction in the cervical spinal cord.^22^ Intra-cisternal magna (ICM) gene delivery might be desirable if it could be performed via i.t. or intravascular catheter^29^^,^^30^; however, considering the accessibility to the ventricles in clinical practice, i.c.v. gene delivery could be the ROA of choice to achieve the desired CNS biodistribution with the maximal neuroprotective efficacy. Additional studies involving gene delivery to effector muscles, including in the diaphragm, need to be assessed in larger animal models as well. Secondly, the pan-neuronal Syn promoter is known to achieve weaker transgene expression in cholinergic SMNs^39^ compared to neurons in the brain. More recent studies have demonstrated that MN-specific promoters exhibit preferential transgene expression in ventral horn MNs throughout the spinal cord when delivered using AAV vectors.^39^^,^^40^^,^^41^^,^^42^ Future studies will be needed to determine whether delivery of MN-specific promoter Cav-1 overexpression using Hb9, eHGT_1137 m pan-SMN enhancer, or *cis*-regulatory element 98 (CRE98)^39^^,^^42^ may provide greater therapeutic efficacy than was observed with *SynCav1* in the current study.

To conclude, the present study demonstrates that i.c.v. *AAV9-SynCav1* gene delivery to hSOD1^G93A^ mice results in improved electrophysiological properties, partially preserved MNs in the cervical and lumbar spinal cord in males, and broad Cav-1 overexpression in the CNS with preservation of NMJs in the diaphragm in both sexes. Based on these findings, i.c.v. administration of *AAV9-SynCav1* may represent a potential strategy for broad CNS delivery of Cav-1 and attenuation of ALS-associated motor system pathology.

## Materials and methods

### Animals

Human mutant SOD-1 (hSOD1^G93A^) Tg mice and C57BL/6 mice were obtained from Jackson Laboratory (Bar Harbor, ME, USA) and bred at our facility. hSOD1^G93A^ male mice were crossed with C57BL/6 females to obtain hSOD1^G93A^-positive mice. Tg-negative mice from the hSOD1^G93A^ strain were used as controls (WT). All mice were treated in compliance with the Guide for the Care and Use of Laboratory Animals (National Institutes of Health, Bethesda, MD, USA). All animal protocols were approved by the Veterans Administration San Diego Healthcare System Institutional Animal Care and Use Committee (IACUC) (#A20-030). Mice were housed on a 12-h:12-h light-dark cycle (lights on at 6 a.m.) in a temperature- and humidity-controlled room under standard conditions with free access to food and water. Mice were housed with littermates (2–4 mice) in standard cages (28.4 × 18.4 × 12.5 cm) floored with corn cob bedding in an individually ventilated caging system.

### Experimental design

The hSOD1^G93A^ mice were randomly assigned to two groups: hSOD1^G93A^ + saline or AAV9-SynRFP (hSOD1^G93A^ + CTRL, *n* = 33) and hSOD1^G93A^ + AAV9-*SynCav1* with Woodchuck hepatitis virus post-transcriptional regulatory element (WPRE) (hSOD1^G93A^ + *SynCav1*, *n* = 32). hSOD1^G93A^ + CTRL consisted of a pooled cohort of mice receiving either saline or AAV9-SynRFP. This pooling strategy was implemented in strict adherence to the 3 Rs (replacement, reduction, and refinement) of ethical animal use, intentionally minimizing the unnecessary breeding and sacrifice of Tg mice. This approach is justified by our prior extensive validation in this exact hSOD1^G93A^ model, which demonstrated that control viral vectors do not alter disease onset, motor decline, anatomical readouts, or survival compared to saline or naive controls, thereby establishing them as biologically and statistically equivalent baselines. Furthermore, to eliminate any potential viral-related confounding variables in key assessments, the control baselines for all male electrophysiological evaluations (MEPs and EMG) and cervical histological cell counts were derived exclusively from saline-injected mice. These groups underwent i.c.v. surgery at the age of 10 weeks, followed by weekly health checks, and an RW test at 18 weeks, with a GS test, MEPs, and EMG performed at 22 weeks of age. WT (*n* = 19) littermates were used as references. The behavioral test operator and data analysts were blinded to the experimental groups until the completion of data analysis. Animals that showed signs of distress and infection at the surgical site were excluded from all above-mentioned behavioral tests. Upon completion of behavioral tests and electrophysiological tests, mice were sacrificed by pentobarbital (100–120 mg/kg) overdose via intraperitoneal injection. Brain, spinal cord, and muscle tissues were collected and processed for biochemical, histopathological, and immunofluorescence analyses. Regarding attrition and exclusions, one hSOD1^G93A^ + *SynCav1*-treated mouse was specifically excluded from the RW and GS behavioral assessments due to skin lesions caused by fighting with cage mates. The overall values vary across assays because specific animal cohorts were sacrificed upon the completion of behavioral and electrophysiological testing to harvest distinct tissues for histological analysis.

### Viral vectors and *SynCav1* construct

The self-complementary AAV (scAAV) construct expressing the neuron-specific Syn promoter was generated together with Cav-1 cDNA by VectorBuilder (Chicago, IL, USA). To address viral titer dilution in the CSF using i.c.v. ROA, a WPRE, which has been proven to enhance AAV transgene property,^20^^,^^21^ was added to the AAV9-*SynCav1* construct. To create the fusion of Syn and Cav-1 cDNA, an XbaI-SalI DNA fragment containing the Syn promoter was inserted into the NheI-SalI site of pEGFP-N1 (Clontech). The resulting plasmid, named pSyn-EGFP, had the 537 bp Cav-1 cDNA excised from the pCRII-TOPO vector (Invitrogen) using PmeI-NotI and inserted into the SmaI-NotI region of pSyn-EGFP, replacing the EGFP gene with Cav-1 cDNA to create pSynCav-1. The Syn-promoter-Cav-1 cassette was then isolated from pSyn-Cav-1. The DNA sequence spanning from the Syn promoter (480 bp) to Cav-1 (537 bp) was synthesized and cloned into the scAAV backbone plasmid, resulting in the scAAV vector construct expressing Cav-1 under the control of the Syn promoter. The scAAV9-*SynCav1* vector was produced by transiently transfecting HEK293T cells with each vector plasmid, pRep2/Cap9, and the pAd-Helper plasmid. Helper virus-free AAV vectors were generated via transient co-transfection of HEK293T cells with three plasmids (pVector, pRep2/CapX [X being the serotype], and pAd-Helper) using polyethylenimine (PEI). Cells were harvested 72 h after transfection, and the cell lysate was prepared through three freeze/thaw cycles. The AAV vectors in the lysate were purified using a combination of sucrose-cushion ultracentrifugation and anion-exchange column chromatography. The virus titer was determined by real-time qPCR, with the number of genome copies (gc/mL) in the vector preparation serving as an indicator of the total genomic content of AAV particles. The final virus titer of the vector reached approximately 7.5 × 10^13^ gc/mL.

### Genotyping

Genotyping of hSOD1^G93A^ mice was performed according to previous procedures.^15^^,^^20^ At 4 weeks of age, tail biopsy samples were collected and digested with 50 mM NaOH at 95°C for 20 min three times. PCR for the *hSOD1* gene was performed as follows: denaturation at 94°C for 2 min; 10 cycles of 94°C for 20 s, 65°C for 15 s (decreasing 0.5°C per cycle), and 68°C for 10 s; 28 cycles of 94°C for 15 s, 60°C for 15 s, and 72°C for 10 s; extension at 72°C for 2 min; and a final hold at 10°C. All primers were purchased from Integrated DNA Technologies (Coralville, IA, USA). *hSOD1*^G93A^-positive mice were confirmed using the following primers: primer 1, *hSOD1* forward (CAT CAG CCC TAA TCC ATC TGA [oIMR0113]); primer 2, *hSOD1* reverse (CGC GAC TAA CAA TCA AAG TGA [oIMR0114]); primer 3, internal control forward (CTA GGC CAC AGA ATT GAA AGA TCT [oIMR7338]); and primer 4, internal control reverse (GTA GGT GGA AAT TCT AGC ATC ATC C [oIMR7339]). Using these primers, an 800 bp product for endogenous mouse *SOD1* and a 600 bp product for human *SOD1* were detected. PCR products were separated on a 1% agarose gel (100 volts for 30 min).

### i.c.v. ROA

All surgeries were performed under sterile conditions and approved by the IACUCs. For i.c.v. injection, mice were anesthetized with 2%–3% isoflurane and kept on a 37°C heating pad during the surgery. The head was fixed in a stereotactic frame, and the surgical site was shaved and disinfected. A midline scalp incision was made, and a burr hole was drilled at coordinates relative to bregma (X = ±1.0 mm, Y = −0.2 mm, Z = −2.0 mm) using a 33G needle. Each mouse received a total of 10 μL of AAV9-*SynCav1* (approximately 7.5 × 10^10^ gc/μL) administered into the CSF space by i.c.v. injection (5 μL per side over 60 s with 2 min of dwelling time). This was performed using a 10 μL Hamilton Gas Tight syringe (Hamilton, Reno, NV, USA) and a digital infusion pump (Microinjector MINJ-PD, Tritech Research). The CTRL group underwent the same craniotomy with either an equal volume of sterile saline or AAV9-SynRFP (2 × 10^10^ gc/μL) injected intracerebroventricularly. For both groups, the skin was closed with 7/0 Vicryl sutures, and analgesic (1 mg/kg slow-release buprenorphine) was administered before recovery from anesthesia.

### Motor tests and electrophysiology

#### RW test

The RW test is used to assess motor dysfunction and the efficacy of *SynCav1* treatment.^15^^,^^20^ At 18 weeks, mice were individually housed in plastic cages measuring 40 × 40 × 35 cm (L × W × H). Each cage was equipped with an 11.5-cm-diameter RW (SuperFlex Open Field, Omnitech Electronics, OH, USA), with free access to food and water and daily health checks. Mice had access to the wheel under a 12-h light/dark cycle (lights on from 6:00 a.m. to 6:00 p.m.). Each experiment lasted 36 h, including two nights, and mice were acclimated to the cages at least 24 h before data collection. Sensor beams connected to a desktop computer via software (Fusion v.6.5 r9999 Super Flex Edition, Omnitech Electronics) recorded wheel rotations, running speed (m/min), and total distance (m), which were used for analysis.

#### GS test

The GS test is reported to reflect limb motor dysfunction in the same mouse model.^43^ Mice were tested for loss of forelimb GS by using the Chatillon Digital Force Gauge (AMETEK, Berwyn, PA, USA) at 22 weeks of age. Mice were held in front of a horizontal bar, such that only the forelimb paws were able to grasp the bar. The mice were gently pulled back with steady force until both paws released the bar. Each mouse was given 6 attempts, and the maximum value was recorded. The mean value of three trials was obtained for each mouse as a GS value.

#### MEPs

MEPs were recorded to assess motor pathway function and disease progression in hSOD1^G93A^ mice.^15^ For electrophysiological measurements, mice were anesthetized with intraperitoneal injection of propofol (100–170 mg/kg). A 30G stimulating electrode was placed subcutaneously over both sides of the motor cortex. For all mice, electrical stimulation was delivered at 0.7 ms duration and 3.14 mA, and responses were recorded from the triceps muscle using 30G needle electrodes (UltraPro S100 EMG/NCS/EP neurodiagnostic system, USA). The distance between recording electrodes was 3–4 mm, and the ground electrode was placed subcutaneously in the tail. After consistently obtaining three waves, the mean latency and amplitude were averaged for each mouse.

#### EMG

To assess motor unit denervation, resting EMG recordings were performed to quantify spontaneous muscle fibrillations and fasciculations.^44^ EMG recordings were subsequently performed after MEPs with 2% isoflurane anesthesia if necessary. Electrodes (30G needles with 5 mm spacing) were inserted into the gastrocnemius muscle. The sampling frequency ranged from 20 Hz to 10 kHz, and data were digitized using LabChart 7 (ADInstruments). Because denervated ALS muscle exhibits spontaneous high-amplitude spikes (fibrillations) at rest,^11^^,^^15^^,^^44^ the integration of the absolute amplitude for 1 s was obtained for each individual mouse and analyzed.

#### Immunoblot assays

The motor cortex, cervical spinal cord (C6–C8), and lumbar spinal cord (L4–L6) were homogenized at 4°C in 500 mM sodium carbonate (pH 11.0) containing protease and phosphatase inhibitors, followed by three rounds of 10 s sonication. After normalizing the concentration, immunoblotting was performed using primary antibodies Cav-1 (Cell Signaling, no. 3268, 1:1,000) and GAPDH (Cell Signaling, no. 2118s, 1:1,000), and the blots were incubated overnight at 4°C. This was followed by a 1-h incubation with HRP-linked anti-rabbit IgG (Cell Signaling Technology, no. 7074S, 1:1,000). Signal was visualized by Lumigen ECL Ultra (Lumigen TMA-6), and densitometry analysis was conducted using Fiji.

#### IF microscopy

After perfusion with phosphate-buffered saline (PBS), tissue was fixed for 48 h in z-fix (ANATECH, #170) and dehydrated in 30% sucrose. Coronal spinal cord sections (C5–C6) were obtained at 30 μm thickness as floating samples. Tissue sections were washed 3 times in Tris-buffered saline (TBS) for 10 min each, and antigen retrieval was performed with citrate antigen retrieval buffer (10 mM citrate and 0.05% Tween 20) at 80°C for 20 min.^16^ Then, tissue sections were left to cool down for 10–15 min. Finally, two additional TBS washes were performed for 5 min each. The sections were then blocked with 5% donkey serum and incubated with Cav-1 (Cell Signaling, no. 3268, 1:1,000), NeuN (Millipore, no. ABN90P, 1:1,000), and ChAT (Millipore, no. AB144P, 1:200) as the primary antibodies overnight at 4°C. A minimum of three coronal sections per mouse was averaged for the quantification of MNs. For the quantification of Cav-1 fluorescence intensity, regions of interest (ROIs) were strictly restricted to ChAT+ MN soma to ensure cell-type-specific analysis. The background Cav-1 intensity from an equivalent area in the adjacent white matter within the same image was subtracted using Fiji. The background-corrected Cav-1 intensity was then normalized to the MN soma area.

For diaphragm muscle staining, 30-μm-thick horizontal diaphragm muscle sections were collected on slides precoated with 1:1 of Sta-On (Leica Biosystems, no. 3803105) and 100% ethanol. After drying at room temperature, slides were washed 3 times in PBS for 10 min each. Next, samples were blocked and permeabilized in 3% bovine serum albumin (BSA) + 3% goat serum diluted in PBS with 0.5% Triton X for 2 h. The sections were then incubated for 2 nights with 488-conjugated α-bungarotoxin (α-BTX) (Invitrogen, no. B13422, 1:250) to label postsynaptic structures and synaptophysin (Abcam, no. ab32594, 1:200) to label presynaptic vesicles, followed by 1-h incubation with secondary Alexa Fluor 555 Goat anti-Rabbit (Thermo Fisher Scientific, no. A-21428, 1:200) at room temperature. The occupancy of synaptophysin-labeled presynaptic structures overlapping with α-BTX-labeled postsynaptic structures was analyzed to assess NMJ morphology.^20^ Slides were imaged using a Keyence All-in-One microscope (BZ-X700, Keyence Corporation of America, IL, USA), and 10 μm z stacks were acquired and projected into single images for analysis using the full-focus image composition function of the BZ-X Analyzer software. NMJ occupancy was defined as the percentage of α-BTX (postsynaptic)-labeled area occupied by synaptophysin (presynaptic) within the projection. Fiji was used for image analyses of both cervical and NMJ assessments.

### RNA extraction and RT-qPCR analysis

#### Tissue collection and RNA extraction

Following transcardial perfusion with PBS to remove circulating blood cells, the muscle tissue was rapidly dissected and stored at −80°C until processing. Total RNA was isolated from diaphragm tissue using the RNeasy Fibrous Tissue Mini Kit (Qiagen, no. 74704) according to the manufacturer’s instructions. RNA concentration and purity were assessed with a spectrophotometer (NanoDrop, ND-1000), and samples with an A260/A280 ratio between 1.8 and 2.1 were used for subsequent analysis.

#### cDNA synthesis

First-strand cDNA was synthesized from 800 ng of total RNA using the iScript Reverse Transcription Supermix (Bio-Rad, no. 1708841) as per the kit protocol. The thermal cycler conditions were 5 min at 25°C, 20 min at 46°C, and 1 min at 95°C.

#### qPCR

qPCR was performed using PowerUp SYBR Green Master Mix (Applied Biosystems, no. A25742) on a StepOnePlus Real-Time PCR System (Applied Biosystems). Each 18 μL reaction contained 8 μL SYBR Green Master Mix, 0.1 μL forward and reverse primers, and 2 μL diluted cDNA (100 ng/μL). The thermal cycling conditions were 10 min at 95°C followed by 40 cycles of 15 s at 95°C and 45 s at 60°C (annealing/extension). A melt curve analysis was performed at the end of each run (from 60°C to 95°C) to confirm the amplification of a single, specific product. Primer information is as follows: *SynCav1*, forward: 5ʹ-GAG GAG TCG TGT CGT GC-3ʹ and reverse: 5ʹ-GGT GTA GAG ATG TCC CTC CG-3ʹ; phosphoglycerate kinase 1 (PGK1), forward: 5ʹ-GAT GCT TTC CGA GCC TCA CTG T-3ʹ and reverse: 5ʹ-ACC AGC CTT CTG TGG CAG ATT C-3ʹ; and synaptophysin, forward: 5ʹ-AGT ACC CAT TCA GGC TGC AC-3ʹ and reverse: 5ʹ-CCG AGG AGG AGT AGT CAC CA-3ʹ. For analysis, *SynCav1* gene expression was normalized to the PGK1 value.

### Statistical analysis

Sample sizes are indicated in each section of the materials and methods. The statistical analysis was performed for experiments with a group size of five or more. Data were analyzed by Student’s *t* test, Welch’s *t* test, Mann-Whitney tests, one-way ANOVA, or two-way ANOVA following Fisher’s LSD, Tukey’s multiple comparison, or Bonferroni’s post hoc analysis with Prism7 software (GraphPad, La Jolla, CA, USA). Fisher’s least significant difference (LSD) test was retained for selected histological endpoints central to our primary biological hypothesis (specifically MN pathology and NMJ denervation), as these comparisons were made among a limited number of predefined experimental groups to explicitly evaluate disease-associated pathology and treatment-associated attenuation. For correlation analysis, simple linear regression with 95% confidence intervals was performed. Data were presented as mean (standard deviation [SD]), and significance was assumed at ∗*p* < 0.05. Each experimental group was blinded, and codes were broken for analysis.

## Data and code availability

The data that support findings of this study are available within this study or available from the corresponding author (B.P.H.) upon reasonable request.

## Acknowledgments

The work was funded by VA RCS BX006318, 10.13039/100000090CDMRP AL210059 (ALSTIA) and AL230115 (ALSTDA), and UCSD Gene Therapy Initiative 2039592.

## Author contributions

N.W. guided the overall study design, wrote the manuscript, performed i.c.v. gene delivery, behavioral tests, IF microscopy, immunoblots, and RT-qPCR. Y.K. performed IF and immunoblots; counted and analyzed MNs; and assisted with animal breeding, genotyping, and behavior tests. V.T. performed IF, analyzed the data, prepared the figures, and revised the manuscript. K.A.O. assisted with genotyping and IF microscopy and revised the manuscript. D.W. provided investigational and i.c.v. surgical guidance and reviewed the manuscript. H.W. provided investigational guidance. N.K. performed the behavioral tests. E.I. provided the scientific concepts and reviewed the manuscript. B.P.H. supervised the project, provided intellectual discussion, edited the manuscript, and funded the project.

## Declaration of interests

B.P.H. holds equity and is a non-paid consultant with Eikonoklastes Therapeutics LLC.

## Declaration of generative AI and AI-assisted technologies in the writing process

During the preparation of this work, the authors used ChatGPT (OpenAI)/Gemini (Google) for language editing, improving English grammar, and refining the academic tone of the manuscript. The authors reviewed and edited the output as needed and take full responsibility for the content of the published article.
